# Effect of species, breed and route of virus inoculation on the pathogenicity of H5N1 highly pathogenic influenza (HPAI) viruses in domestic ducks

**DOI:** 10.1186/1297-9716-44-62

**Published:** 2013-07-22

**Authors:** Mary Pantin-Jackwood, David E Swayne, Diane Smith, Eric Shepherd

**Affiliations:** 1 Exotic and Emerging Avian Viral Diseases Unit, Southeast Poultry Research Laboratory, USDA-Agricultural Research Service, 934 College Station Road, Athens, GA 30605, USA

## Abstract

H5N1 highly pathogenic avian influenza (HPAI) viruses continue to be a threat to poultry in many regions of the world. Domestic ducks have been recognized as one of the primary factors in the spread of H5N1 HPAI. In this study we examined the pathogenicity of H5N1 HPAI viruses in different species and breeds of domestic ducks and the effect of route of virus inoculation on the outcome of infection. We determined that the pathogenicity of H5N1 HPAI viruses varies between the two common farmed duck species, with Muscovy ducks (*Cairina moschata*) presenting more severe disease than various breeds of *Anas platyrhynchos var. domestica* ducks including Pekin, Mallard-type, Black Runners, Rouen, and Khaki Campbell ducks. We also found that Pekin and Muscovy ducks inoculated with two H5N1 HPAI viruses of different virulence, given by any one of three routes (intranasal, intracloacal, or intraocular), became infected with the viruses. Regardless of the route of inoculation, the outcome of infection was similar for each species but depended on the virulence of the virus used. Muscovy ducks showed more severe clinical signs and higher mortality than the Pekin ducks. In conclusion, domestic ducks are susceptible to H5N1 HPAI virus infection by different routes of exposure, but the presentation of the disease varied by virus strain and duck species. This information helps support the planning and implementation of H5N1 HPAI surveillance and control measures in countries with large domestic duck populations.

## Introduction

Domestic ducks play an important role in the epidemiology of H5N1 highly pathogenic avian influenza (HPAI) viruses in Asia, Africa and Eastern Europe. When using the chicken intravenous pathogenicity index test (IVPI), the international standard pathotyping test, H5N1 HPAI viruses are by definition highly lethal to chickens; however, in domestic ducks these viruses can produce a range of clinical outcomes from asymptomatic infections to severe disease with mortality [1-7]. Both sick and asymptomatic infected ducks can shed high virus quantities into the environment favoring increased risk of transmission and potential outbreaks in commercial chickens and threatening human health. Infected migratory waterfowl including various duck species, most prominently the Mallard (*Anas platyrhynchos*) are suspected of contributing to the spread of H5N1 HPAI viruses from Asia to other parts of the world [8-10]. However, the penetration and circulation of H5N1 HPAI viruses in domestic duck populations is considered to be one of the major sources of infection with these viruses, thus perpetuating the enzootic cycle of H5N1 HPAI in several countries in free-range farmed, as well as backyard or village-reared domestic ducks [10-13]. Domestic ducks are often farmed in open fields, flooded rice paddies, or on ponds or other bodies of water, this farming approach allowing direct exposure to wild waterfowl and domestic ducks from multiple duck farmers, providing many mechanisms for introductions or spread of virus between farms [14]. In addition to direct contact with infected birds, contamination of the environment with viruses shed by infected ducks plays an important role in the indirect transmission of avian influenza (AI) viruses to susceptible birds [15-17]. Water has for a long time been suspected as source of AI virus to infect migratory waterfowl [18-22], and AI virus has been experimentally shown to be transmitted from infected Mallard to naïve ducks through a common source of water [23].

There are many types or breeds of ducks that are farmed, but most domestic ducks are descendants of the wild Mallard (*Anas platyrhynchos)*[24]. The other major domestic duck species is the Muscovy (*Cairina moschata*), which was domesticated in its native South America, but has spread throughout the world including Asia and Europe via agricultural production [25]. The species of the two domestic ducks, as well as with different wild duck species, has been shown to affect the outcome of H5N1 HPAI infection, with some duck species being more likely to show clinical signs and higher mortality after virus infection [5,26,27]. In a previous study we found clear differences in the pathogenicity and response to vaccination against H5N1 HPAI (HA clade 2.3.4) between Pekin (*Anas platyrhynchos, var. domestica*) and Muscovy ducks [28]. In a second study we examined infection with a different strain of H5N1 HPAI virus (HA clade 1), in Muscovy, Pekin, and a Mallard-type duck phenotypically closer to wild Mallard and again, Muscovy ducks showed more severe clinical signs and mortality than either of the *Anas* sp. ducks [29]. However, no studies have been conducted comparing the infectivity and pathogenicity of H5N1 HPAI viruses between multiple breeds of *Anas platyrhynchos var. domestica* domestic ducks.

The differences observed in pathogenicity of H5N1 HPAI viruses in domestic ducks has implications in surveillance and control of the disease, as asymptomatic or mildly symptomatic infected ducks are difficult to recognize and can spread the virus to other susceptible poultry. In the present study we build on our previous results and explore both the effect of species and of duck breed on the pathogenicity of H5N1 HPAI, and also study the effect of different routes of virus inoculation by infecting Muscovy and Pekin ducks with two H5N1 HPAI viruses of different virulence.

## Materials and methods

### Viruses

The following H5N1 HPAI viruses were used in this study: A/bar-headed goose/Mongolia/X53/2009 (HA clade 2.3.2.1) (Mongolia/09) (Courtesy of Malik Peiris, Hong Kong University and Martin Gilbert, Wildlife Conservation Society), A/Ck/Egypt/9402NAMRU3-CLEVB213/2007 (Egypt/07) (HA clade 2.2.1.1) and A/CK/Egypt/08124S-NLQP/2008 (Egypt/08) (HA clade 2.2.1). The latter two viruses were used in a previous study [30]. The viruses were propagated in embryonating chicken eggs (ECE) as previously described [31]. Allantoic fluid was diluted in brain heart infusion (BHI) medium (BD Bioscience, Sparks, MD) in order to obtain an inoculum with 10^6^ 50% egg infectious dose (EID_50_) per 0.1 mL/bird. A sham inoculum was made using sterile allantoic fluid diluted 1:300 in brain heart infusion (BHI) medium (BD Bioscience, Sparks, MD). All experiments using H5N1 HPAI viruses, including work with animals, were performed in biosecurity level-3 enhanced (BSL-3E and ABSL-3E) facilities at the Southeast Poultry Research Laboratory (SEPRL), Agricultural Research Service, United States Department of Agriculture (USDA), and all personnel were required to wear a powered air purifying respirator with high efficiency particulate air (HEPA)-filtration (3M™, St. Paul, MN, USA).

### Ducks

A single representative of Muscovy ducks (*Cairina moschata*) and five breeds of *Anas platyrhynchos var. domestica* domestic ducks (Pekin, Mallard-type, Black Runners, Rouen, and Khaki Campbell) were obtained at one day of age from commercial farms and maintained at SEPRL facilities. Mallard-types are considered phenotypically and genetically most close to wild Mallards and are also sold and maintained as domestic ducks, primarily for hunter release in North America. At two weeks of age, ducks were housed in self-contained isolation units that were ventilated under negative pressure with HEPA-filtered air and maintained under continuous lighting. Serum samples were collected from ten ducks from each breed prior to beginning the experiments to ensure that the birds were serologically negative for AI viruses ELISA (FlockCheck Avian Influenza MultiS-Screen Antibody Test^®^, IDEXX Laboratories, Westbrook, ME, USA). Feed and water were provided with ad libitum access. All bird experiments were approved and performed under the regulations of the SEPRL Institutional Animal Care and Use Committee.

### Pathogenicity studies

Two similar experiments were conducted. The first experiment examined the effect of species and breed of ducks on the pathogenicity of a H5N1 HPAI virus. The second experiment examined the effect of the route of virus inoculation on the pathogenicity of two different H5N1 HPAI viruses in Muscovy and Pekin ducks.

#### ***Study 1: effect of species and breed of ducks***

The pathogenicity of Mongolia/09 H5N1 HPAI virus was examined in *Cairina moschata* (Muscovy) and *Anas platyrhynchos var. domestica* (five breeds: Muscovy, Pekin, Mallard-type, Black Runners, Rouen, and Khaki Campbell ducks). The experimental design has been previously described [30,32]. Briefly, two-week-old ducks of each breed were separated into controls groups and virus-inoculated groups. The control groups contained 10 ducks and these were intranasally (IN) inoculated through the choanal cleft with 0.1 mL of sham inoculum. The virus-inoculated groups, each also containing 10 ducks, were inoculated IN with inoculum containing 10^6^ EID_50_ of the viruses in 0.1 mL. Two birds from each group were euthanized at 2 days post-inoculation (dpi) and the following tissues were collected in 10% neutral buffered formalin solution to determine microscopic lesions and the extent of virus replication in tissues: trachea, lung, heart, brain, adrenal gland, proventriculus, duodenum, jejunum, ceca, pancreas, liver, kidney, spleen, bursa, thymus, Harderian gland, tongue, and feathered skin, and skeletal muscle from the left thigh. Samples were prepared as previously described [3,33]. Portions of the brain, lung, skeletal muscle, heart and spleen were also collected in BHI containing an antibiotic/antimycotic mixture. The remaining birds were observed for clinical signs over a 9 day period during which time any clinical signs were recorded. At 3 dpi, body temperatures via rectal thermometer, and oropharyngeal and cloacal swabs were collected. Ducks with severe neurological signs, that stopped eating or drinking, or remained recumbent, were euthanized and counted dead as for the next day. Sample birds, moribund birds, with severe neurological signs or were unable to reach food or water, and all birds remaining at the end of the 9-day period were euthanized by the intravenous (IV) administration of sodium pentobarbital (100 mg/kg body weight).

#### ***Study 2: effect of route of virus inoculation***

Groups of 10 two-week-old Muscovy ducks (*Cairina moschata)* and Pekin ducks (*Anas platyrhynchos var. domestica)* were inoculated via intranasal (IN), intracloacal (IC) or intraocular (IO) with 10^6^ EID_50_ in 0.1 mL of Egypt/07 or Egypt/08 H5N1 HPAI viruses. These two viruses had previously shown to have different levels of virulence in Pekin ducks [30]. The pathogenicity of these two viruses hasn’t been determined in chickens, but similar viruses produced 100% mortality within days of infection as expected for HPAI viruses in chickens [2]. Two control groups containing 10 ducks of each species were IN inoculated with 0.1 mL of sham inoculum. Two birds from each group were euthanized at 2 dpi and the tissues collected to determine microscopic lesions; portions of the brain, lung, skeletal muscle, heart and spleen were taken for virus titration. Clinical signs of the remaining ducks were recorded over a 9 day period. Body temperatures and oropharyngeal and cloacal swabs for virus detection and quantification were collected from all ducks at 3 dpi.

### Virus titrations

Tissues collected in BHI were stored at -70 °C until use. Titers of infectious virus were determined by weighing, homogenizing tissues, and diluting in BHI to a 10% (wt/vol) concentration. Ten-fold dilutions of the 10% homogenates (100 μL) were inoculated into 10 day old ECE and virus titers as log 10 EID_50_/gram of tissue were calculated [31]. The threshold of detection for virus titers in tissues was 10^1.97^ EID_50_/g of tissue.

Oropharyngeal and cloacal swabs were collected in 2 mL of BHI medium with 1× antibiotic/antimycotic and kept frozen at -70 °C until RNA extraction. RNA was extracted using a previously described combination of Trizol LS reagent (Invitrogen Inc. Carlsbad, CA, USA) and the MagMax AI/ND RNA isolation kit (Ambion, Inc. Austin, TX, USA) [34]. Quantitative real time RT-PCR (qRT-PCR) was performed as previously described [35] with modifications. Briefly, qRT-PCR targeting the influenza M gene was conducted using AgPath-ID one-step RT-PCR Kit (Ambion, Austin, TX, USA) and the ABI 7500 Fast Real-Time PCR system (Applied Biosystem, Carlsbad, CA, USA). A standard curve for virus quantification was established with RNA extracted from dilutions of the same titrated stock of the challenge virus, and results reported as EID_50_/mL equivalents [36]. The calculated qRRT-PCR lower detection limit was 10^1.6^ EID_50_/mL per reaction.

### Statistical analyses

Data were analyzed using Prism v.5.01 software (GraphPad Software Inc.) and values are expressed as the mean ± SD. The survival rate data was analyzed using the Mantel-Cox Log-Rank test. One-way ANOVA with Tukey post-test was used to analyze body temperatures and virus titers in swabs. For statistical purposes, all oropharyngeal and cloacal swabs from which virus were not detected were given a numeric value of 10^1.6^ EID_50_/mL. These values represent the lowest detectable level of virus in these samples based on the methods used. Statistical significance was set at *p* < 0.05.

## Results

### Study 1: effect of species and breed on the pathogenicity of a H5N1 HPAI virus in domestic ducks

**Figure 1:**
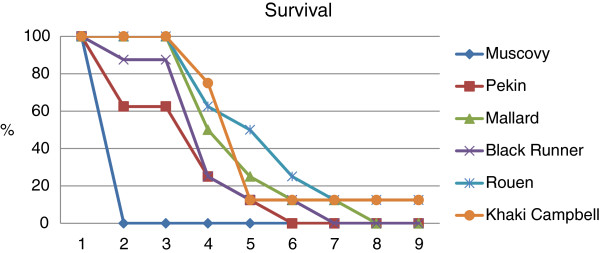
**Study 1: survival rate of ducks after inoculation with the Mongolia/09 H5N1 HPAI virus.** Two-week-old ducks were inoculated with 10^6^ EID_50_ of the virus and mortality was monitored for 9 days. The length of survival was statistically shorter for Muscovy ducks (*Cairina moschata*) compared to the five breeds of *Anas platyrhynchos var. domestica*, as determined by the log rank test (*p* < 0.05).

The outcome of intranasal inoculation of different species and breeds of two-week-old ducks with Mongolia/09 H5N1 HPAI is shown in Figure 1. Infection with the virus resulted in 100% mortality in 4 groups of ducks and only the Rouen and Khaki Campbell groups having one survivor. All Muscovy ducks died by 2 dpi, while the five breeds of *Anas platyrhynchos var. domestica* had mean death times (MDT) of over 3.6 days for which each was significantly longer than MDT for Muscovy ducks. All *Anas platyrhynchos var. domestica* ducks showed a significant increase in body temperatures at 3 dpi when compared with the controls for each group (see Additional file 1). No significant difference in body temperature was observed between the virus-infected Pekin, Mallards, Black Runners, Rouen and Khaki Campbell ducks (*p* < 0.05). Muscovy ducks showed only severe lethargy before death. One to five ducks from the five breeds of *Anas platyrhynchos var. domestica* presented neurological signs including tremors, loss of balance, tilted head, loss of vision, seizures, and paralysis (see Additional file 1). The average onset of neurological signs was 3 dpi. Ducks in all these groups were also listless, had anorexia and watery greenish diarrhea. Two ducks (one Rouen and one Khaki Campbell) presented no or mild to moderate listlessness and recovered by the end of the 9 day period. No clinical signs were observed in the sham-inoculated control ducks.

Two ducks per group were necropsied and examined at 2 dpi. No gross lesions were observed in the sham-inoculated ducks. All virus-infected ducks presented with non-specific gross lesions including dehydration, empty intestines, splenomegaly, thymic atrophy, dilated and flaccid hearts with increased pericardial fluid, and congested malacic brains, as reported in previous studies [2]. Microscopic lesions were widespread in tissues from all ducks examined and were similar to previously described for H5N1 HPAI virus infections in domestic ducks [2,3]. The most consistent lesions were moderate to severe rhinitis and sinusitis, mild to moderate tracheitis and bronchitis, mild to severe interstitial pneumonia, mild to moderate multifocal necrosis of cardiac myofibers, and in the brain, randomly scattered foci of malacia with gliosis. Also commonly observed was mild multifocal pancreatitis, necrosis of the epithelia of the Harderian glands, and mild to moderate multifocal areas of vacuolar degeneration to necrosis of the corticotrophic cells of the adrenal gland. Mild to moderate necrosis of hepatocytes with sinusoidal histiocytosis was observed in the liver. The spleen, thymus, bursa, and mucosa-associated lymphoid tissue had mild to moderate lymphoid depletion ranging from apoptosis to necrosis in remaining lymphocytes. No microscopic lesions were present in tissues from the sham-inoculated ducks.

Viral antigen staining was present in multiple tissues of all ducks infected with the virus, indicating systemic infection (see Additional file 2). Viral antigen was observed in the pancreatic acinar epithelium, neurons and glial cells of the brain, trachea epithelium, alveolar epithelium, fragmented cardiac and skeletal myofibers, adrenal corticotrophic cells, and Harderian gland epithelium. In lymphoid organs, viral antigen was only identified in resident and infiltrating phagocytes. Viral antigen was also identified in the glandular epithelium of the proventriculus, in hepatocytes and Kupffer cells in the liver, smooth muscle of the ventriculus, autonomic ganglia of the enteric tract, and feather epidermal cells. Virus infection in Muscovy ducks resulted in more widespread and intense viral antigen staining in tissues compared to the five breed of *Anas platyrhynchos var*. *domestica* ducks.

**Figure 2:**
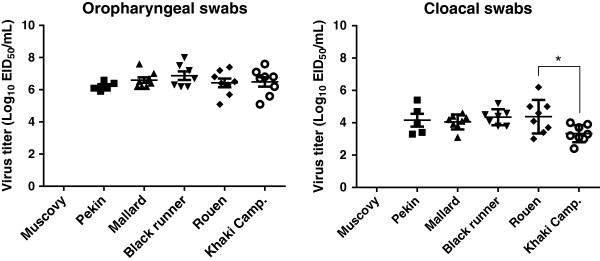
**Study 1: virus shedding.** Ducks were intranasally inoculated with the Mongolia/09 H5N1 HPAI virus. Oropharyngeal and cloacal swabs were taken from all birds remaining at 3 dpi. For Muscovy ducks, no shedding data was available because all died by 2 dpi. Groups with asterisk are significantly different (*p* < 0.05).

Moderate to high virus titers (log10 ^5.1–7.8^ EID_50_) were present in the lung, brain, heart, spleen, and muscle of virus-challenged ducks corroborating the systemic viral replication (see Additional file 3). Shedding of virus through the oropharyngeal and cloacal routes was examined at 3 dpi by rRT-PCR (Figure 2). No results are available for the Muscovy ducks because they were all dead by 2 dpi. No significant differences in oropharyngeal and cloacal virus shedding was observed between the different breed of *Anas platyrhynchos var. domestica* ducks, with the exception of cloacal shedding between Rouen and Khaki Campbell, with Khaki Campbell ducks shedding less virus (*p* < 0.05).

### Study 2: effect of route of virus inoculation

**Figure 3:**
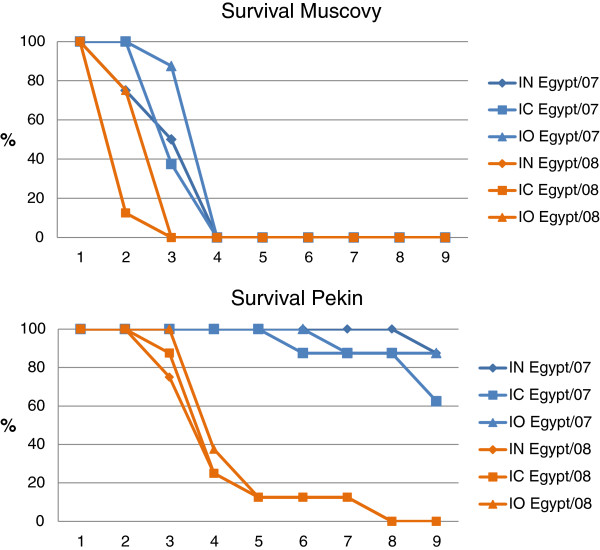
**Study 2: survival rates of Muscovy (*Cairina moschata*) and Pekin (*Anas platyrhynchos var. domestica*) ducks inoculated by the intranasal (IN), intracloacal (IC), or intraocular (IO) routes with the Egypt/07 or Egypt/08 H5N1 HPAI viruses.** Survival rates were not statistically different between the ducks receiving virus by the same route of inoculation as determined by the log rank test (*p* < 0.05). Survival rates were not different between all Muscovy ducks regardless of the virus, with the exception of ducks inoculated with Egypt/07 via IO and ducks inoculated with Egypt/08 via IC. Survival rates were different between Pekin ducks inoculated with Egypt/07 compared with Egypt/08.

The result of intranasal (IN), intracloacal (IC), or intraocular (IO) inoculation of Muscovy (*Cairina moschata*) and Pekin (*Anas platyrhynchos var. domestica*) ducks with one of two different H5N1 HPAI viruses is shown in Figure 3.

### Muscovy ducks

Inoculation of the Muscovy ducks with Egypt/07 or Egypt/08 resulted in 100% mortality regardless of the virus or the route of inoculation used. MDT was longer for ducks inoculated through the IO route. However, survival rates were not significantly different when comparing all groups of Muscovy ducks regardless of the virus or the route of inoculation, with the exception of ducks inoculated with Egypt/07 via IO and ducks inoculated with Egypt/08 via IC which were different (*p* < 0.05). Most ducks were listless, had anorexia and diarrhea by 2 dpi; however some ducks died without presenting clinical signs. Four to seven of eight Muscovy ducks inoculated with Egypt/07 presented neurological signs starting at 3 dpi, some of them sick enough to require euthanasia. Two or six ducks from the groups inoculated with Egypt/08 had neurological signs at 2 dpi, but most were found dead at this time point. No significant differences were observed in body temperatures of the surviving infected Muscovy ducks when compared to the control ducks at 3 dpi, with the exception of the Muscovy ducks inoculated IN with Egypt/07 which did have significantly higher body temperatures than controls (see Additional file 4).

No gross lesions were observed in the 2 sham-inoculated ducks necropsied and examined at 2 dpi. Muscovy ducks infected with either virus showed the characteristic lesions of infection with H5N1 HPAI viruses including dehydration, empty intestines, splenomegaly, thymic atrophy, dilated and flaccid hearts with increased pericardial fluid, and congested malacic brains. No difference in gross lesions was observed between the different groups of ducks. Microscopic lesions were widespread in tissues from all ducks examined and were similar to previously described for virulent H5N1 HPAI [2,3] and described in Study 1. Viral antigen staining was present in most tissues examined, and was more widespread in ducks infected with Egypt/08 given by any of the three routes of inoculation (see Additional file 5). Viral staining was more evident in some tissues than others depending on the route of inoculation, with intestine, gonads, cloacal bursa and pancreas showing stronger viral staining in ducks infected via IC route; and eye, eyelid, and Harderian gland in ducks inoculated via IO route (see Additional files 5 and 6). In any case, the virus was present in many tissues indicating early dissemination with systemic infection.

**Figure 4:**
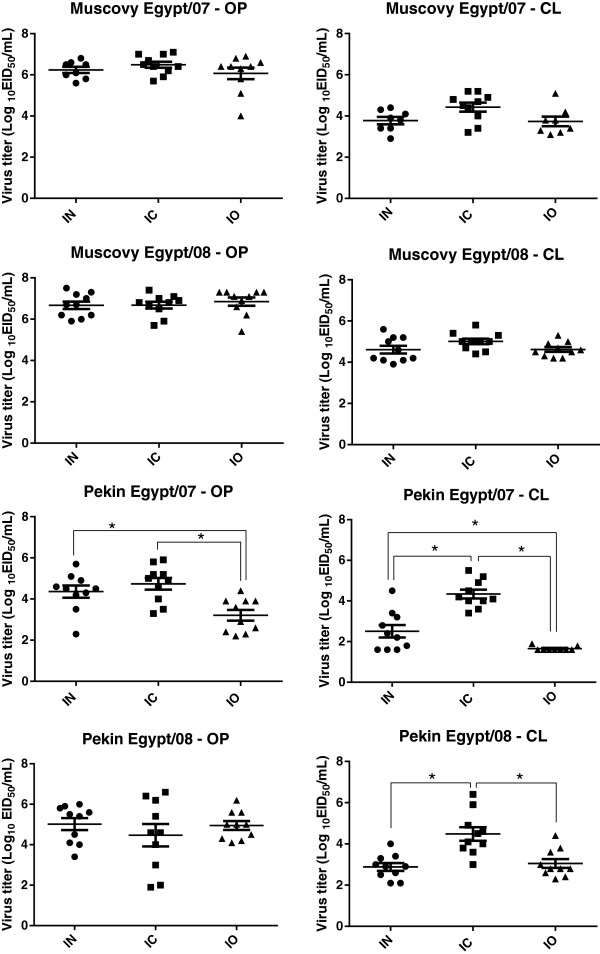
**Study 2: virus shedding.** Ducks were inoculated by the intranasal (IN), intracloacal (IC), or intraocular (IO) route with Egypt/07 or Egypt/08 H5N1 HPAI virus. Oropharyngeal and cloacal swabs were taken from all remaining birds at 3 dpi. Groups with asterisk (*) are significantly different (*p* < 0.05).

Moderate to high virus titers (log10 ^4–8.3^ EID_50_) were present in the lung, brain, heart, spleen, and muscle of Muscovy ducks challenged by either virus given by any of the three routes of inoculation (see Additional file 7). Shedding of virus through the oropharyngeal and cloacal routes was examined at 2 dpi by rRT-PCR (Figure 4). Results included swabs from ducks found dead at that time point. No significant differences in oropharyngeal and cloacal virus shedding were found between Muscovy ducks given the viruses by the different routes (*p* < 0.05). However, in general, titers from ducks inoculated with Egypt/08 where higher than those inoculated with Egypt/07.

### Pekin ducks

Clear differences in mortality were observed when comparing Pekin ducks inoculated with the Egypt/07 with ducks inoculated with Egypt/08 (Figure 3). Only one to three ducks died, with similar MDTs, when inoculated with Egypt/07, the highest mortality found in ducks inoculated by the IC route. Inoculation of Pekin ducks with Egypt/08 resulted in 100% mortality regardless of the virus or the route of inoculation used, however a longer MDT was observed in ducks inoculated by the IO route (7 dpi) compared to ducks inoculated by the IN (5.1 dpi) or IC (5.3 dpi) route. Survival rates were not statistically different between Pekin ducks receiving virus by the same route of inoculation, as determined by the log rank test (*P* < 0.05), but were different between Pekin ducks inoculated with Egypt/07 compared to Pekin ducks inoculated with Egypt/08. Only one or three of eight ducks inoculated with Egypt/07 presented neurological signs and lethargy and eventually died. The rest of the ducks showed no clinical signs. In all three groups of ducks inoculated with Egypt/08, six of eight ducks showed neurological signs and all were listless, and had anorexia and diarrhea. Significant differences in body temperatures were observed at 3 dpi when compared to the control ducks, with the exception of the Pekin ducks inoculated IN with Egypt/07 controls (see Additional file 4). Body temperatures were generally higher in ducks inoculated with Egypt/08.

No gross or microscopic lesions were observed in the 2 sham-inoculated ducks and in the 2 ducks belonging to all three groups inoculated with Egypt/07 when examined at 2 dpi. Minimal or no virus staining was observed in tissues from these ducks at this time point controls (see Additional file 8). Ducks infected with Egypt/07 by the IC route had some virus staining in lymphoid tissues, not observed in ducks inoculated by the other two routes. Virus titers in lung, heart, spleen and brain were also higher for ducks inoculated by the IC route (see Additional file 7). There were differences in virus shedding (*p* < 0.05) between the three different groups (Figure 4). Oropharyngeal shedding was higher in ducks inoculated by the IN and IC route when compared to ducks inoculated by the IO route. Cloacal shedding was higher in ducks inoculated by the IC route compared to ducks inoculated by the IN and IO route, and ducks inoculated by the IN route shed more virus than ducks inoculated by the IO route.

Pekin ducks infected with Egypt/08 showed the characteristic gross lesions of infection with virulent H5N1 HPAI viruses but no differences were observed between the different groups of ducks. Microscopic lesions were present in most tissues from the ducks examined and were similar to previously described for virulent H5N1 HPAI viruses [2,3]. However virus antigen staining was more widespread in tissues from ducks inoculated by the IC route (see Additional file 8). Moderate to high virus titers (log10 ^2.2–7.3^ EID_50_) were present in the lung, brain, heart, spleen, and muscle of Pekin ducks challenged with Egypt/08 given by any of the three routes of inoculation (see Additional file 7), the lowest titers found in the heart of ducks inoculated by the IC route. Oropharyngeal virus shedding was similar for all three groups, but cloacal virus shedding was significantly higher for ducks inoculated by the IC route (*p* < 0.5).

## Discussion

In our previous studies we found clear differences in the pathogenicity of H5N1 HPAI viruses between Muscovy ducks (*Cairina moschata*) and Pekin ducks (*Anas platyrhynchos var. domestica)*, with Muscovy ducks presenting a more severe disease after infection than Pekin ducks [28,29]. In this study we expanded on our previous findings by also examining for differences in disease presentation not only between the two species of domestic ducks, *Cairina moschata* and *Anas platyrhynchos var. domestica*, but also in different breeds of *Anas platyrhynchos var. domestica*. Pekin, Black Runner, Rouen, and Khaki Campbell ducks, although all considered descendants of the wild Mallard are very different in many aspects including appearance, temperament and purpose (meat versus egg production). In this study we found that ducks from the same species but different breed respond similarly to infection with a lethal H5N1 HPAI virus. However, minor differences between breeds were observed. Mallard, Black Runner, Rouen and Khaki Campbell ducks survived for longer after infection with the H5N1 HPAI virus than Pekin ducks, but not significantly; one Rouen and one Khaki Campbell duck survived virus infection; and Khaki Campbell’s shed less virus by the cloacal route than Rouen ducks. These differences in pathogenicity might become more evident if a less pathogenic strain of H5N1 HPAI is used as challenge virus or if a lower virus titer is given [37]. We also found that Muscovy and Pekin ducks became infected with two H5N1 HPAI viruses of different virulence when given by any one of three routes, intranasal (IN), intracloacal (IC), or intraocular (IO). Regardless of the route of inoculation, the outcome of infection was similar for each species and depended on the virulence of the virus used. Infection with either virus was lethal to all Muscovy ducks, however only one of the viruses caused high mortality in Pekin ducks, again stressing the clear differences in pathogenicity of H5N1 HPAI viruses in these two duck species.

Until 2002, H5N1 HPAI viruses caused only mild or no clinical disease in ducks. Since then, many H5N1 HPAI viruses have shown to be pathogenic in ducks [2], but the pathogenicity of these viruses depends on the virus strain, the age of the ducks, and, as this and other studies demonstrate, the duck species. Differences in virus pathogenicity among duck species has been previously reported for domestic and wild ducks [4,5,26-28]. Based on the results of our study, there is a clear difference in response to H5N1 HPAI virus infection between Muscovy ducks (*Cairina moschata*) when compared to the *Anas platyrhynchos var. domestica* ducks. All ducks presented high mortality after challenge with the Mongolia/09 H5N1 HPAI virus and although high virus titers were found in tissues of all ducks examined at 2 dpi, demonstrating that all were equally susceptible to infection, the *Anas platyrhynchos var. domestica* ducks survived virus infection for longer than the Muscovy ducks. In the second study, Muscovy and Pekin ducks also became systemically infected with the two different H5N1 HPAI viruses, but all Muscovy ducks succumbed to infection with either virus while most Pekin ducks survived infection with one of the viruses (Egypt/07). As previously reported, there are clear differences in pathogenicity between H5N1 HPAI viruses in domestic ducks, with some viruses being more virulent than others. However, as shown in this second experiment, these differences can become more evident depending on the duck species. Similar to the first experiment, systemic virus replication was demonstrated in all ducks; however the virus titers in the Pekin ducks, especially for the Egypt/07 virus, were in general lower than the observed in the Muscovy ducks. Previous studies have also shown that Pekin ducks present a less severe disease after challenge with H5N1 HPAI virus than Muscovy ducks, and also mount a stronger humoral immune response to vaccination [28,29]. The reason for the differences observed in H5N1 HPAI virus pathogenicity between the two species can be in part explained by differences in the immune responses between ducks. Avian influenza virus infection induces a cascade of host defenses that are responsible for control and clearance of the virus and includes innate and subsequent adaptive immune responses. *Anas platyrhynchos var. domestica* ducks might be more efficient in controlling virus replication and spread after infection than Muscovy ducks and consequently, able to clear the virus and survive the infection, or survive for longer [29]*.*

Domestic ducks might become infected by different routes with H5N1 HPAI viruses. Different from low pathogenic avian influenza (LPAI) viruses, H5N1 HPAI viruses replicate preferentially in the respiratory tract of ducks, yet still replicate in the intestinal tract, and virus is excreted in high titers in both feces and respiratory or oral secretions [2]. Most studies examining the pathogenicity of H5N1 HPAI viruses in ducks have used the intranasal (IN) route of inoculation [5,27,28,32,38-44]. However, other routes of exposure have been used to experimentally infect ducks. Trying to emulate natural exposure, ducks were infected by inoculating virus simultaneously via the cloaca, trachea, throat, nares and eyes [7,45,46]. Simultaneous inoculation by the IN and intraocular (IO) routes, or IN, IO and through the mouth, has also been used to infect ducks with H5N1 HPAI virus [1,47,48], and wild ducks were infected with a H5N1 HPAI virus after simultaneous inoculation by the intratracheal and intraesophageal routes [9]. Infection with a H5N1 HPAI virus caused morbidity and mortality in domestic ducks after ingestion of infected meat and inoculation by the intragastric and IN routes [49]. Ducks also became infected after ingestion of feathers with H5N1 HPAI virus [42]. The route of virus exposure has been shown to be important in causing AI in other avian species. Turkeys and chickens can be infected AI viruses by the IC and intraoviduct routes [50-53]. Cloacal exposure has also been shown to be important to the transmission of other pathogens in poultry. For example, cloacal contact with feces that have been contaminated with *Histomonas meleagridis* is thought to be one way in which blackhead is transmitted from bird to bird [54], and intra-cloacal inoculation or vaccination has been used experimentally with other viruses [55].

Recently, it was shown that Mallards ducks can be infected with LPAI viruses by various routes of inoculation with very similar pattern of viral shedding [56]. It’s not known if H5N1 HPAI viruses can infect equally well ducks by different routes of exposure, and if infection by different routes will result in differences in the presentation of disease. As demonstrated in our study, H5N1 HPAI viruses readily infect ducks through other routes besides the IN route. Not only did the ducks become infected when challenged by the IC and IO route, but presented very similar clinical disease, gross and histological lesions and virus replication staining in tissues as the IN-infected ducks. This suggests that irrespective of the initial site of replication, the virus rapidly becomes systemic and produces similar lesions and grows to similar high titers in tissues. In the Muscovy ducks, both Egypt/07 and Egypt/08 viruses, no matter by which route they were given, replicated to high titers in tissues and induced almost identical lethal diseases. However, in the Pekin ducks, infection with the two viruses resulted in very different outcomes. One virus produced similar lethal systemic disease to that in the Muscovy ducks, while the other virus only killed one to three ducks. Nevertheless, all ducks became infected with the viruses regardless of the route of exposure and the virus given. Another difference observed in the Pekin ducks was that ducks infected through the IC route shed more virus through the cloaca than ducks inoculated by the IN or IO route.

LPAI viral transmission in aquatic bird populations is thought to occur through an indirect fecal-oral route involving contaminated water [18,21,22]. In experimental trials it has been demonstrated that unlike wild-type LPAI viruses, replication of the H5N1 HPAI viruses in ducks is primarily associated with the respiratory tract. However fecal shedding does occur and contact transmission has been demonstrated under experimental conditions [18]. Ducks can shed H5N1 HPAI virus via the cloacal and respiratory routes for many days [46]. Infected ducks can contaminate ponds, fields or wetlands they inhabit with H5N1 HPAI viruses which can survive in these environments for variable lengths of time [11,57]. Most *Anas platyrhynchos var. domestica* domestic ducks are “dabblers”, which tend to feed superficially (skimming the surface of water for feed), but can also feed on and filter mud in shallow waterways [58]. Ducks in water are also allegedly practice “cloacal sipping” (in which water is sucked into the cloaca), which could potentially enhance spread of infection if the water is contaminated with virus [58]. The fate of respiratory-borne virus from ducks in water is not known. Since ducks are gregarious animals, the shift towards increased excretion of H5N1 HPAI virus via the respiratory route could potentially facilitate duck-to-duck transmission when birds are in close contact [58]. However, studies of rates of transmission between ducks for viruses excreted predominantly via the cloacal or oropharyngeal route remain to be conducted. Understanding how ducks become infected with H5N1 HPAI virus will help improve husbandry practices to prevent disease outbreaks.

In conclusion, we demonstrated that domestic ducks are susceptible to H5N1 HPAI virus infection by different routes of exposure, but the presentation of the disease will vary depending on the virus strain and the duck species, with only minor differences between breeds of ducks. This information will help in understanding how H5N1 HPAI transmits in domestic ducks and how it can present in different ways in different species.

## Competing interests

The authors declare they have no competing interests.

## Authors’ contributions

MPJ conceived the studies, coordinated the work described, performed the necropsies, the histopathology and immunohistochemistry, analyzed the data and wrote the manuscript. DES was involved in the experimental design, interpretation of results and critically read the manuscript. DS and ES prepared the viruses, helped conduct the animal studies, and processed and helped analyze the samples (virus isolation and RRT-PCR). All authors read and approved the final manuscript.

## Supplementary Material

Additional file 1 **Study 1.** Body temperature, rate of neurological signs and mortality. Two-week-old ducks were intranasally inoculated with the Mongolia/09 H5N1 HPAI virus. Click here for file

Additional file 2 **Study 1.** Distribution of viral antigen in tissues collected from ducks intranasally inoculated with the Mongolia/09 H5N1 HPAI virus. Tissues were collected from 2 ducks at 2 days post challenge. Click here for file

Additional file 3 **Study 1.** virus titers in tissues collected from ducks intranasally inoculated with the Mongolia/09 H5N1 HPAI virus. Tissues were collected at 2 dpi from 2 ducks. Values are the means ± standard deviation. The threshold of detection was 10^1.97^ EID_50_/gram. Click here for file

Additional file 4 **Study 2.** Body temperature, rate of neurological signs and mortality. Two-week-old Muscovy (*Cairina moschata*) and Pekin (*Anas platyrhynchos var. domestica*) ducks were inoculated by the intranasal (IN), intracloacal (IC), or intraocular (IO) routes with Egypt/07 or Egypt/08 H5N1 HPAI viruses. Click here for file

Additional file 5 **Study 2.** Distribution of viral antigen in tissues collected from Muscovy ducks (*Cairina moschata*) inoculated by the intranasal (IN), intracloacal (IC), or intraocular (IO) route with Egypt/07 or Egypt/08 H5N1 HPAI virus. Tissues were collected at 2 dpi from 2 ducks. Click here for file

Additional file 6 **Study 2.** Immunohistochemical staining for avian influenza virus antigen in tissues of Muscovy ducks (*Cairina moschata*) infected with Egypt/08 H5N1 HPAI virus, 2 dpi. Viral antigen (in red) in epithelial cells of the eye cornea (A, 400X); in epithelial cells of the ciliary processes of the eye (B, 400X); in the epithelial cells of the Harderian gland (C, 400X); and in histiocytes and necrotic cellular debris in the bursa (D, 400X). Click here for file

Additional file 7 **Study 2.** Virus titers in tissues collected from ducks inoculated by the intranasal (IN), intracloacal (IC), or intraocular (IO) route with Egypt/07 or Egypt/08 H5N1 HPAI virus. Tissues were collected at 2 dpi from 2 ducks. Values are the means ± standard deviation. The threshold of detection was 10^1.97^ EID_50_/gram. Click here for file

Additional file 8 **Study 2.** Distribution of viral antigen in tissues collected from Pekin ducks (*Anas platyrhynchos var. domestica*) inoculated by the intranasal (IN), intracloacal (IC), or intraocular (IO) route with Egypt/07 or Egypt/08 H5N1 HPAI virus. Tissues were collected at 2 dpi from 2 ducks. Click here for file
