# Supplementary material for: Effect of species, breed and route of virus inoculation on the pathogenicity of H5N1 highly pathogenic influenza (HPAI) viruses in domestic ducks
Source: Vet Res. 2013 Jul 22;44(1):62. doi: 10.1186/1297-9716-44-62 (PMC3733953; doi:10.1186/1297-9716-44-62)
Supplement: Additional file 1 — Study 1. Body temperature, rate of neurological signs and mortality. Two-week-old ducks were intranasally inoculated with the Mongolia/09 H5N1 HPAI virus. [file 1297-9716-44-62-S1.docx]

| Groups | Body temperatures at 3dpi (°F)^A^ | # ducks with neurological signs/total  (day of onset) | Mortality/ total (mean death time in days) |
| --- | --- | --- | --- |
| Muscovy controls | 106.1 ± 0.3^a^ | 0/8 | 0/8 |
| Muscovy infected | 106.0 ^a^ | 0/8 | 8/8 (2) |
| Pekin controls | 107.0 ± 0.6 ^a^ | 0/8 | 0/8 |
| Pekin infected | 109.2 ± 0.3^b^ | 4/8 (2) | 8/8 (3.6) |
| Mallard controls | 106.9 ± 0.2 ^a^ | 0/8 | 0/8 |
| Mallard infected | 108.5 ± 0.9 ^b^ | 5/8 (3) | 8/8 (4.9) |
| Black runner controls | 107.2 ± 0.5 ^a^ | 0/8 | 0/8 |
| Black runner infected | 108.5 ± 0.6 ^b^ | 3/8 (2) | 8/8 (4.1) |
| Rouen control | 105.7 ± 0.2 ^a^ | 0/8 | 0/8 |
| Rouen infected | 108.6 ± 0.4 ^b^ | 1/8 (4) | 7/8 (4.1) |
| Khaki Campbell controls | 106.9 ± 0.9 ^a^ | 0/8 | 0/8 |
| Khaki Campbell infected | 108.9 ± 0.5 ^b^ | 3/8 (4) | 7/8 (4.7) |

^A^Mean ± SD. Groups with different lowercase are significantly different (*p*<0.05)
