# Supplementary material for: Effect of species, breed and route of virus inoculation on the pathogenicity of H5N1 highly pathogenic influenza (HPAI) viruses in domestic ducks
Source: Vet Res. 2013 Jul 22;44(1):62. doi: 10.1186/1297-9716-44-62 (PMC3733953; doi:10.1186/1297-9716-44-62)
Supplement: Additional file 2 — Study 1. Distribution of viral antigen in tissues collected from ducks intranasally inoculated with the Mongolia/09 H5N1 HPAI virus. Tissues were collected from 2 ducks at 2 days post challenge. [file 1297-9716-44-62-S2.docx]

| Tissue | Muscovy  (*Cairina moschata*) | *Anas platyrhynchos* *var.* *domestica* | | | | |
| --- | --- | --- | --- | --- | --- | --- |
|  |  | Pekin | Mallard-type | Black runner | Rouen | Khaki Campbell |
| Nasal | ++/++^A^ | +/+ | +/+ | +/+ | +/+ | +/+ |
| Trachea | +/+ | +/+ | +/+ | +/+ | +/+ | +/+ |
| Lung | +++/++ | +/+++ | +/++ | ++/+ | +/+ | ++/+ |
| Heart | +++/+ | ++/+ | ++/++ | ++/+ | +/+ | ++/+ |
| Eye lid | +/+++ | ++/++ | ++/+ | +/+ | +/+ | +/+ |
| Harderian gland | +++/+ | ++/+ | +/+ | +/+ | -/+ | +/- |
| Thymus | ++/++ | +/++ | -/- | -/- | -/- | +/- |
| Bursa | +/+ | -/- | -/- | -/- | -/- | -/- |
| Spleen | +/- | -/- | -/+ | -/- | -/- | +/- |
| Liver | +/- | -/++ | +/+ | -/- | -/- | -/+ |
| Intestine | -/- | -/- | -/- | -/- | -/- | -/- |
| Pancreas | ++/- | +/- | -/- | +/- | -/+ | -/- |
| Gonads | ++/- | +/- | -/- | +/- | -/+ | -/- |
| Brain | ++/+ | +/+ | ++/+ | ++/+ | ++/+ | ++/+ |
| Feather | -/- | -/- | -/- | -/- | -/- | -/- |
| Muscle | ++/- | +/+ | -/+ | +/+ | -/+ | +/- |
| Adrenals | ++/- | +/+++ | +/- | +/+ | -/++ | ++/- |

^A^ Duck 1/duck 2. - + no virus antigen staining; + = infrequent; ++ = common; +++ =widespread.
