# Supplementary material for: Effect of species, breed and route of virus inoculation on the pathogenicity of H5N1 highly pathogenic influenza (HPAI) viruses in domestic ducks
Source: Vet Res. 2013 Jul 22;44(1):62. doi: 10.1186/1297-9716-44-62 (PMC3733953; doi:10.1186/1297-9716-44-62)
Supplement: Additional file 4 — Study 2. Body temperature, rate of neurological signs and mortality. Two-week-old Muscovy (Cairina moschata) and Pekin (Anas platyrhynchos var. domestica) ducks were inoculated by the intranasal (IN), intracloacal (IC), or intraocular (IO) routes with Egypt/07 or Egypt/08 H5N1 HPAI viruses. [file 1297-9716-44-62-S4.docx]

| Duck species | Virus and | Route of inoculation | Body temperatures at 3 dpi (°F)^A^ | # ducks with neurological signs/total  (day of onset) | Mortality/ total (mean death time in days) |
| --- | --- | --- | --- | --- | --- |
| Muscovy | Sham inoculum | IN | 106.7 ± 0.5^a^ | 0/8 | 0/8 |
|  | Egypt/07 | IN | 108.2 ± 0.6^bc^ | 6/8 (3) | 8/8 (3.3) |
|  |  | IC | 107.9 ± 1.0^ac^ | 4/8 (3) | 8/8 (3.3) |
|  |  | IO | 107.8 ± 1.0^ac^ | 7/8 (3) | 8/8 (3.8) |
|  | Egypt/08 | IN | - | 2/8 (2) | 8/8 (2.1) |
|  |  | IC | 108.1± 0.1^bc^ | 2/8 (2) | 8/8 (2.1) |
|  |  | IO | 108.1 ± 0.9^bc^ | 6/8 (2) | 8/8 (2.5) |
| Pekin | Sham inoculum | IN | 106.8 ± 0.7 ^a^ | 0/8 | 0/8 |
|  | Egypt/ 07 | IN | 107.3 ± 1.1^ab^ | 1/8 (9 dpi) | 1/8 (9 dpi) |
|  |  | IC | 108.1 ± 1.0^bd^ | 3/8 (6 dpi) | 3/8 (8 dpi) |
|  |  | IO | 108.4 ± 0.8 ^be^ | 1/8 (6 dpi) | 1/8 (7dpi) |
|  | Egypt/08 | IN | 109.3 ± 0.5^ce^ | 6/8 (4 dpi) | 8/8 (5.1 dpi) |
|  |  | IC | 109.1 ± 0.8^cd^ | 6/8 (4 dpi) | 8/8 (5.3 dpi) |
|  |  | IO | 109.4 ± 0.4^c^ | 6/8 (4 dpi) | 8/8 (7 dpi) |

^A^Mean ± SD. Groups within species with different lowercase are significantly different (*p*< 0.05)
