# Supplementary material for: Effect of species, breed and route of virus inoculation on the pathogenicity of H5N1 highly pathogenic influenza (HPAI) viruses in domestic ducks
Source: Vet Res. 2013 Jul 22;44(1):62. doi: 10.1186/1297-9716-44-62 (PMC3733953; doi:10.1186/1297-9716-44-62)
Supplement: Additional file 5 — Study 2. Distribution of viral antigen in tissues collected from Muscovy ducks (Cairina moschata) inoculated by the intranasal (IN), intracloacal (IC), or intraocular (IO) route with Egypt/07 or Egypt/08 H5N1 HPAI virus. Tissues were collected at 2 dpi from 2 ducks. [file 1297-9716-44-62-S5.docx]

| Tissue | Egypt/07 | | | Egypt/08 | | |
| --- | --- | --- | --- | --- | --- | --- |
|  | IN | IC | IO | IN | IC | IO |
| Nasal | +/+ ^A^ | +/++ | +/+ | ++/++ | ++/++ | ++/++ |
| Trachea | -/+ | -/+ | +/- | +/+ | +/+ | +/+ |
| Lung | +++/+ | +++/+++ | +++/++ | +++/+++ | +++/+++ | +++/+++ |
| Heart | +/+ | +/++ | ++/+ | ++/+++ | +++/+++ | ++/+++ |
| Eye lid | +/- | +/+ | +/++ | ++/++ | +/++ | +++/+++ |
| Harderian gland | ++/- | +/+ | ++/++ | ++/+ | +/+ | ++/++ |
| Thymus | +/+ | +/++ | -/- | +++/+++ | +++/+++ | +++/+++ |
| Bursa | ++/- | ++/++ | +/- | +++/+ | +++/+++ | ++/+++ |
| Spleen | -/- | ++/+ | +/- | ++/+ | ++/++ | ++/++ |
| Liver | -/- | +/+ | ++/- | +++/+++ | +++/+++ | +++/+++ |
| Kidney | -/- | -/+ | ++/- | +/+ | +/+ | +/+ |
| Intestine | -/- | ++/++ | ++/- | ++/++ | +++/+++ | +++/+++ |
| Pancreas | -/++ | +/++ | +/- | +++/+++ | +++/+++ | +++/+++ |
| Gonads | -/- | ++/+ | ++/- | +/+ | +/+ | +/+++ |
| Eye | -/- | -/- | -/- | -/- | -/- | ++/++ |
| Brain | +/++ | +/++ | +++/+ | +++/+++ | +++/+++ | +++/+++ |
| Feath.follicle | +/+ | +/+ | -/- | ++/++ | ++/+ | ++/++ |
| Muscle | -/- | +/- | +/+ | +/+ | ++/+ | +/+ |
| Adrenals | +/+ | +/+++ | ++/- | +++/++ | ++/++ | ++/+ |

^A^Duck 1/duck 2. - + no virus antigen staining; + = infrequent; ++ = common; +++ =widespread.
