# Supplementary figures and images for: Effect of species, breed and route of virus inoculation on the pathogenicity of H5N1 highly pathogenic influenza (HPAI) viruses in domestic ducks
Source: Vet Res. 2013 Jul 22;44(1):62. doi: 10.1186/1297-9716-44-62 (PMC3733953; doi:10.1186/1297-9716-44-62)

**
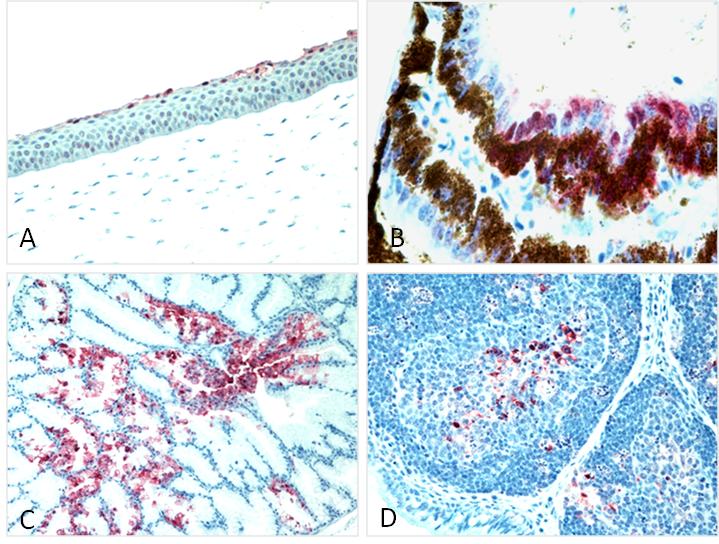
**

Supplement: Additional file 6 — Study 2. Immunohistochemical staining for avian influenza virus antigen in tissues of Muscovy ducks (Cairina moschata) infected with Egypt/08 H5N1 HPAI virus, 2 dpi. Viral antigen (in red) in epithelial cells of the eye cornea (A, 400X); in epithelial cells of the ciliary processes of the eye (B, 400X); in the epithelial cells of the Harderian gland (C, 400X); and in histiocytes and necrotic cellular debris in the bursa (D, 400X). [file 1297-9716-44-62-S6.docx]
