# Supplementary material for: Effect of species, breed and route of virus inoculation on the pathogenicity of H5N1 highly pathogenic influenza (HPAI) viruses in domestic ducks
Source: Vet Res. 2013 Jul 22;44(1):62. doi: 10.1186/1297-9716-44-62 (PMC3733953; doi:10.1186/1297-9716-44-62)
Supplement: Additional file 8 — Study 2. Distribution of viral antigen in tissues collected from Pekin ducks (Anas platyrhynchos var. domestica) inoculated by the intranasal (IN), intracloacal (IC), or intraocular (IO) route with Egypt/07 or Egypt/08 H5N1 HPAI virus. Tissues were collected at 2 dpi from 2 ducks. [file 1297-9716-44-62-S8.docx]

| Tissue | Egypt/07 | | | Egypt/08 | | |
| --- | --- | --- | --- | --- | --- | --- |
|  | IN | IC | IO | IN | IC | IO |
| Nasal | -/+^A^ | -/- | -/- | +/+ | +/+ | +/+ |
| Trachea | -/- | +/++ | -/- | +++/- | +/+ | +/+ |
| Lung | -/- | -/- | +/- | ++/+++ | +/+++ | ++/++ |
| Heart | -/- | -/- | -/- | +/- | +/++ | +/+ |
| Eye lid | -/- | -/- | -/- | ++/++ | +/+ | -/- |
| Harderian gland | -/- | -/++ | -/- | -/++ | -/+++ | -/- |
| Thymus | -/- | +/+ | +/- | +/++ | ++/+++ | +/- |
| Bursa | -/- | +/+ | -/- | -/+ | +/+ | -/- |
| Spleen | -/- | -/+ | -/- | -/+ | ++/++ | +/+ |
| Liver | -/- | -/- | -/- | -/++ | +/++ | +/+ |
| Kidney | -/- | -/- | -/- | -/- | -/+ | -/- |
| Intestine | -/- | -/- | -/- | -/- | -/- | -/- |
| Pancreas | -/- | -/- | -/- | +/++ | +/+++ | -/+ |
| Gonads | -/- | -/- | -/- | -/- | -/+ | +/- |
| Eye | -/- | -/- | -/- | -/- | -/- | -/- |
| Brain | -/- | +/- | -/- | -/+ | +/++ | -/- |
| Hair follicle | -/- | -/- | -/- | -/- | -/- | -/- |
| Muscle | -/- | -/- | -/- | -/- | -/++ | -/- |
| Adrenals | -/- | -/- | -/- | +/+ | -/++ | +/- |

^A^Duck 1/duck 2. - + no virus antigen staining; + = infrequent; ++ = common; +++ =widespread.
