# Supplementary material for: Effect of species, breed and route of virus inoculation on the pathogenicity of H5N1 highly pathogenic influenza (HPAI) viruses in domestic ducks
Source: Vet Res. 2013 Jul 22;44(1):62. doi: 10.1186/1297-9716-44-62 (PMC3733953; doi:10.1186/1297-9716-44-62)
Supplement: Supplementary file 7 [file 1297-9716-44-62-S3.docx]

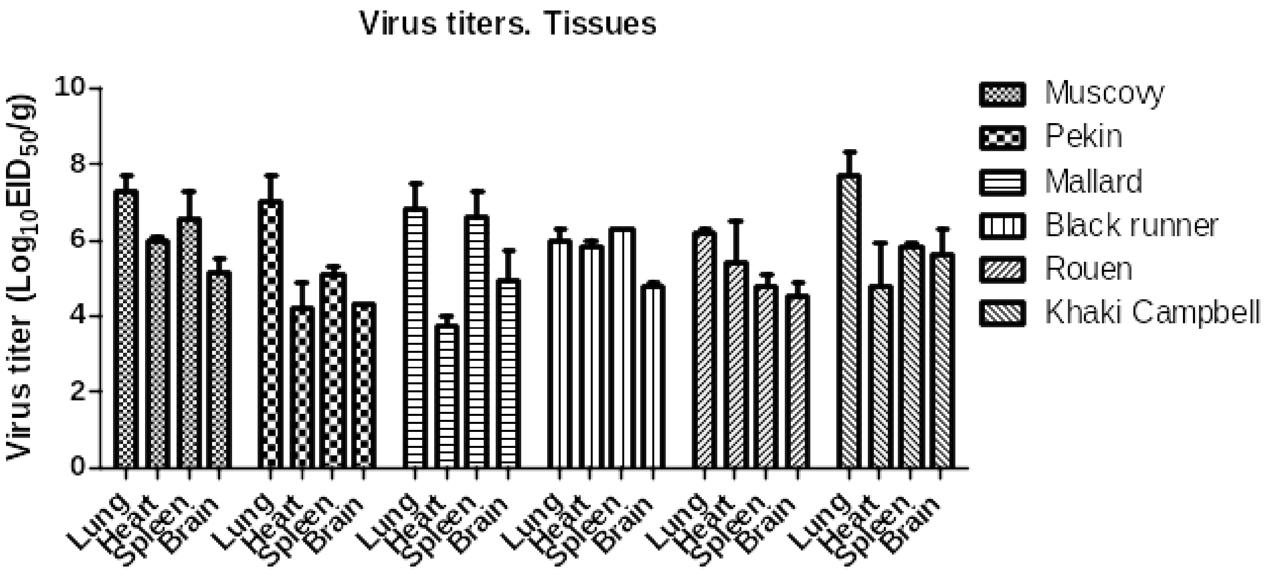

Virus titers. Tissues
Virus titer (Log10EID50/g)
10
8
6
4
2
0
Lung
Heart
Spleen
Brain
Muscovy
Pekin
Mallard
Black runner
Rouen
Khaki Campbell
