# Supplementary material for: Effect of species, breed and route of virus inoculation on the pathogenicity of H5N1 highly pathogenic influenza (HPAI) viruses in domestic ducks
Source: Vet Res. 2013 Jul 22;44(1):62. doi: 10.1186/1297-9716-44-62 (PMC3733953; doi:10.1186/1297-9716-44-62)
Supplement: Supplementary file 8 [file 1297-9716-44-62-S7.docx]

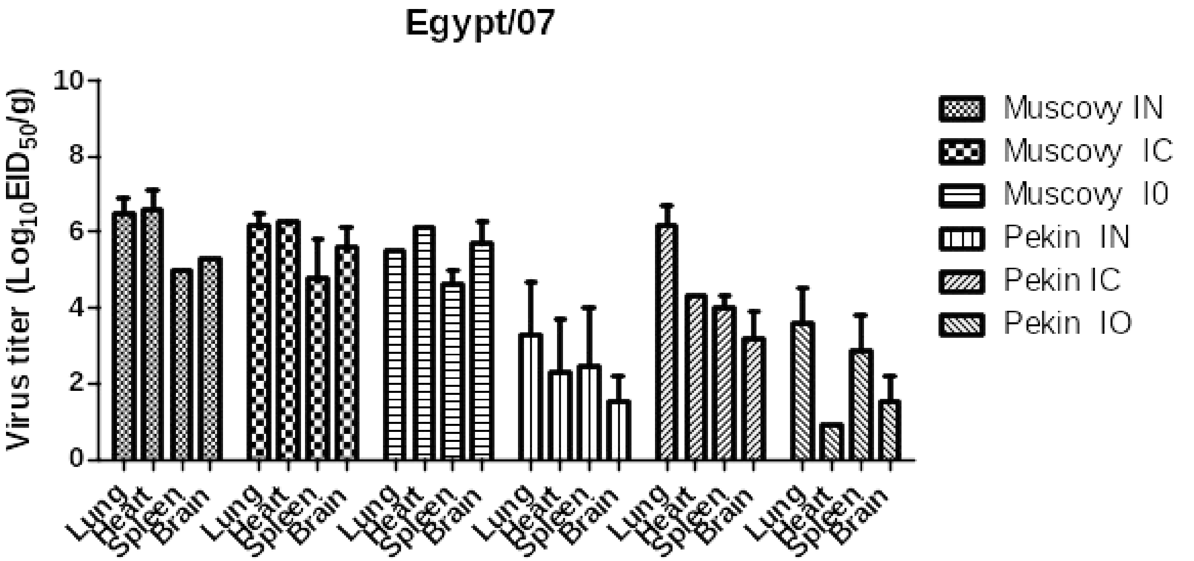
Egypt/07
Virus titer ($Log_{10}EID_{50}/g$)
0
2
4
6
8
10
Lung
Heart
Spleen
Brain
Muscovy IN
Muscovy IC
Muscovy I0
Pekin IN
Pekin IC
Pekin IO

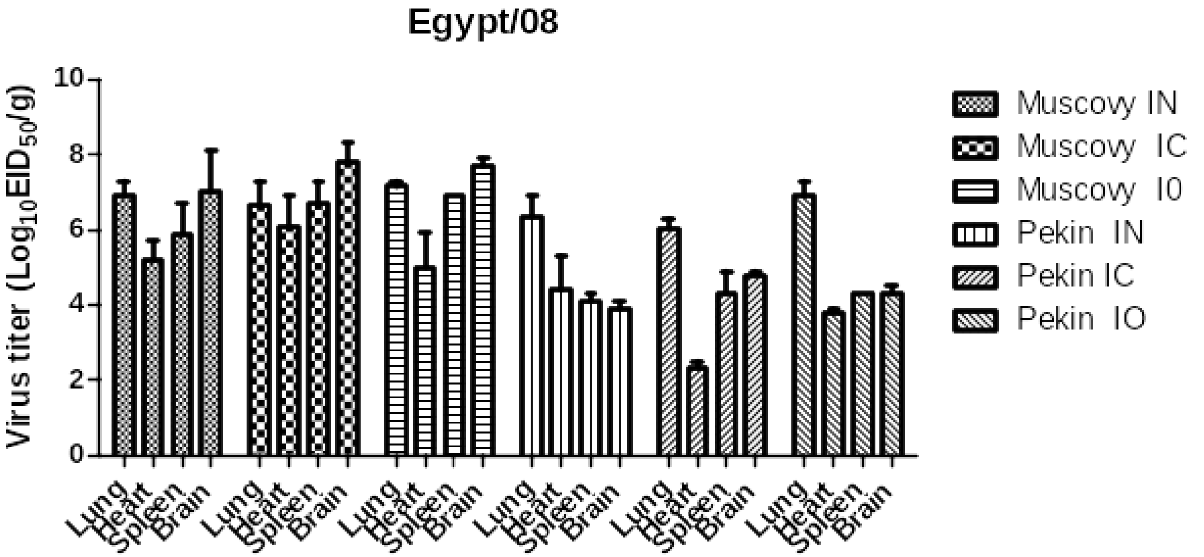
Egypt/08
Virus titer ($Log_{10}EID_{50}/g$)
0
2
4
6
8
10
Lung
Heart
Spleen
Brain
Muscovy IN
Muscovy IC
Muscovy I0
Pekin IN
Pekin IC
Pekin IO
